# Robotic-arm assisted medial unicondylar knee arthroplasty versus jig-based unicompartmental knee arthroplasty with navigation control: study protocol for a prospective randomised controlled trial

**DOI:** 10.1186/s13063-020-04631-5

**Published:** 2020-08-17

**Authors:** Babar Kayani, Sujith Konan, Jenni Tahmassebi, Atif Ayuob, Peter D. Moriarty, Fares S. Haddad

**Affiliations:** Department of Trauma and Orthopaedic Surgery, University College Hospital, 235 Euston Road, Fitzrovia, London, NW1 2BU UK

## Abstract

**Background:**

There remains a paucity of clinical studies assessing how any differences in accuracy of implant positioning between robotic-arm assisted unicompartmental knee arthroplasty (RO UKA) and conventional jig-based unicompartmental knee arthroplasty (CO UKA) translate to patient satisfaction, functional outcomes, and implant survivorship. The objectives of this study are to compare accuracy of implant positioning, limb alignment, patient satisfaction, functional outcomes, implant survivorship, cost-effectiveness, and complications in CO UKA versus RO UKA. Computer navigation will be used to assess intraoperative knee kinematics in all patients undergoing CO UKA.

**Methods and analysis:**

This prospective randomised controlled trial will include 140 patients with symptomatic medial compartment knee arthritis undergoing primary UKA. Following informed consent, patients will be randomised to CO UKA (control group) or RO UKA (investigation group) at a ratio of 1:1 using an online random number generator. The primary objective of this study is to compare accuracy of implant positioning in CO UKA versus RO UKA. The secondary objectives are to compare the following outcomes between the two treatment groups: limb alignment, surgical efficiency, postoperative functional rehabilitation, functional outcomes, quality of life, range of motion, resource use, cost effectivness, and complications. Observers will review patients at regular intervals for 2 years after surgery to record predefined study outcomes pertaining to these objectives. Ethical approval was obtained from the London-Bloomsbury Research Ethics Committee, UK. The study is sponsored by University College London, UK.

**Discussion:**

This study compares a comprehensive and robust range of clinical, functional, and radiological outcomes in CO UKA versus RO UKA. The findings of this study will provide an improved understanding of the differences in CO UKA versus RO UKA with respect to accuracy of implant positioning, patient satisfaction, functional outcomes, implant survivorship, cost-effectiveness, and complications.

**Trial registration:**

ClinicalTrials.gov NCT04095637. Registered on 19 September 2019.

## Background

Unicompartmental knee arthroplasty (UKA) is an established and highly effective treatment for patients with end-stage arthritis affecting a single compartment of the knee joint [2]. The procedure accounts for between 8 and 10% of all knee arthroplasty procedures performed in the UK [1, 15]. There are several advantages of performing UKA over total knee arthroplasty (TKA), including reduced operating time, decreased intraoperative blood loss, reduced periarticular soft tissue trauma, improved preservation of bone stock, better restoration of native kinematics, increased patient satisfaction, and improved functional outcomes [7, 16, 20, 21, 34, 35]. However, UKA is associated with decreased implant survivorship and increased revision rates compared with TKA [9, 28]. Accuracy of component positioning and limb alignment are important prognostic variables that affect implant survival and time to revision surgery following UKA [5, 9, 38]. Consequently, techniques that improve the accuracy of implant positioning and limb alignment in UKA may help to improve long-term survivorship and reduce the burden of revision disease. Conventional jig-based UKA (CO UKA) is performed using manually positioned alignment guides and cutting blocks, limited intraoperative data on knee kinematics, and handheld milling devices or sawblades for bone resection. These techniques are highly dependent on the skill and expertise of the operating surgeons [32, 34]. Studies using data from three separate national joint registries have demonstrated a relationship between the surgical (or unit) case-load and revision rate following UKA [29–32]. Surgeon-controlled errors in implant positioning are the most common reason for implant failure, and low case-volume has been identified as a risk factor for early revision surgery following UKA [30, 32].

Evolution in surgical technology has led to the development of robotic-arm assisted UKA (RO UKA). This uses a preoperative computerised tomography (CT) scan to create a patient-specific virtual three-dimensional reconstruction of the knee joint. The surgeon uses this virtual model to plan optimal bone coverage, implant positioning, and limb alignment for each patient’s unique knee anatomy. An intraoperative robotic arm then helps to execute this plan with a high-level of accuracy, and stereotactic boundaries limit bone resection to the predefined femoral and tibial haptic windows [4, 26, 37]. Intraoperative optical motion capture technology provides real-time medial and lateral gap measurements whilst applying valgus/varus strains to appropriately tension the ligaments through the arc of flexion [10, 24, 26]. Intraoperative data on the ‘tightness’ and ‘looseness’ of the knee joint through the arc of flexion may be used to further adjust bone resection, implant sizes, and implant positions to achieve the desired knee kinematics. Aseptic loosening and progression of osteoarthritis in the remaining native knee compartments are common reasons for failure in UKA [8, 15, 16]. RO UKA enables accurate intraoperative assessment of limb alignment to avoid overcorrection, which may reduce disease progression in the remaining native compartments and help to improve overall implant survivorship. Improved accuracy of bone resection within the confines of the predefined stereotactic boundaries may also reduce periarticular soft tissue injury and enhance postoperative rehabilitation compared to CO UKA [25, 37]. Initial studies have shown that RO UKA is associated with improved accuracy of implant positioning, reduced outliers in limb alignment, faster postoperative rehabilitation, earlier time to hospital discharge, and improved implant survivorship compared to CO UKA [4, 6, 10, 26, 27, 36].

Despite these promising preliminary results with RO UKA, there remains a paucity of high-quality studies comparing a comprehensive and robust range of clinical, functional, and radiological outcomes to CO UKA. Cobb et al. conducted a prospective randomised study on 27 patients with medial compartment knee osteoarthritis undergoing CO UKA versus RO UKA [10]. The authors reported that all patients undergoing RO UKA had tibiofemoral alignment in the coronal plane within 2° of the planned position compared with only 40% in those undergoing CO UKA. Bell et al. performed a prospective randomised controlled study assessing accuracy of implant positioning using postoperative CT scans in 62 RO UKAs versus 58 CO UKAs and found that RO UKA reduced root mean square errors in achieving planned femoral and tibial implant positioning [4]. Blyth et al. followed these patients and found RO UKA was associated with reduced median pain scores by 55.4% compared with CO UKA from postoperative day 1 to week 8 after surgery [6]. RO UKA was associated with improved American Knee Society Score for 3 months following surgery, but there was no difference in functional outcomes observed between CO UKA and RO UKA at 1 year after surgery. Subgroup analysis of the 35 most active patients revealed robotic UKA improved Knee Society Scores, Oxford Knee Scores, and Forgotten Joint Scores compared with CO UKA at 2 years’ follow-up. Herry et al. reviewed plain radiographs in 40 CO UKAs versus 40 RO UKAs and found improved restitution of the native joint line with robotic-guided surgery [18]. Kayani et al. conducted a prospective cohort study on 146 patients showing RO UKA was associated with reduced postoperative pain, decreased opiate analgesia consumption, reduced need for inpatient physiotherapy sessions, and decreased mean time to hospital discharge compared with CO UKA [27]. The main limitations of the aforementioned studies were that outcomes were recorded by non-blinded observers, surgery was undertaken by multiple surgeons with varying experience in UKA, and only limited clinical or functional outcomes were presented at short-term follow-up.

The delayed uptake of RO UKA has been attributed to the substantive installation and maintenance costs of this technology and limited data showing any clinical or functional benefit to an already well-established and successful CO UKA [4, 6, 8, 15, 26, 34, 35]. Pearle et al. conducted a prospective, multicentre review of 1135 RO UKAs and found implant survivorship was 98.8% at a minimum of 22 months’ follow-up, which is superior to the survival rates of CO UKA reported in the national joint registries of the UK (95.6%), Sweden (95.3%), Australia (95.1%), and New Zealand (96.1%) [36]. Batailler et al. compared outcomes in 80 CO UKAs versus 80 RO UKAs and found revision rates in RO UKA were 5% compared with 9% in CO UKA, although this difference was not statistically significant [3]. Moschetti et al. used a Markov decision analysis tool to compare cost-effectiveness of CO UKA versus RO UKA [33]. Using a 2-year failure rate of 1.2% for RO UKA and 3.1% for CO UKA, the authors reported that RO UKA was a cost-effective procedure only if RO UKA case-volume exceeded 94 cases per year. The main limitations of this study were that it did not include the additional costs associated with purchasing the robotic device, buying new implants compatible with the robotic computer software, additional training for the surgical team, increased operative times during the learning phase, and patient resource use. There remains a paucity of data on how RO UKA impacts functional outcomes, quality-adjusted-life years (QALYs), implant survivorship, and cumulative revision rates compared to CO UKA.

In the proposed study, we aim to improve on existing trials by assessing a more comprehensive and robust range of functional and radiological outcomes, prospectively randomising patients to their treatment groups, using standardised surgical techniques in each treatment group, and collecting data at regular follow-up intervals after surgery. This study aims to build on the previous trials by Bell et al. and Blyth et al. by using three-dimensional preoperative templating in both treatment groups, inserting navigation pins to assess knee kinematics and limb alignment in CO UKA, assessing a more comprehensive range of functional outcome scores, blinding patients and observers recording clinical outcomes, and recording study outcomes for a robust analysis of cost-effectiveness and resource use between the two treatment groups [4, 6, 14]. The findings of this study will enable an improved understanding of differences in CO UKA versus RO UKA with respect to patient satisfaction, functional outcomes, implant survivorship, cost-effectiveness, and complications.

## Methods/design

### Objectives

The primary objective of this study is to compare accuracy of implant positioning in CO UKA versus RO UKA. Accuracy of implant positioning will be assessed by measuring differences in the planned implant position on preoperative CT scan versus the achieved implant position on postoperative CT scan. The null hypothesis is that there is no difference in accuracy of implant positioning between CO UKA versus RO UKA.

The secondary objectives are to compare the following outcomes between the two treatment groups:

1. Limb alignment
2. Surgical efficiency
3. Postoperative functional rehabilitation
4. Functional outcomes
5. Quality of life
6. Range of motion
7. Mobilisation distance
8. Resource use and cost effectiveness
9. Complications

### Trial design

**Fig. 1:**
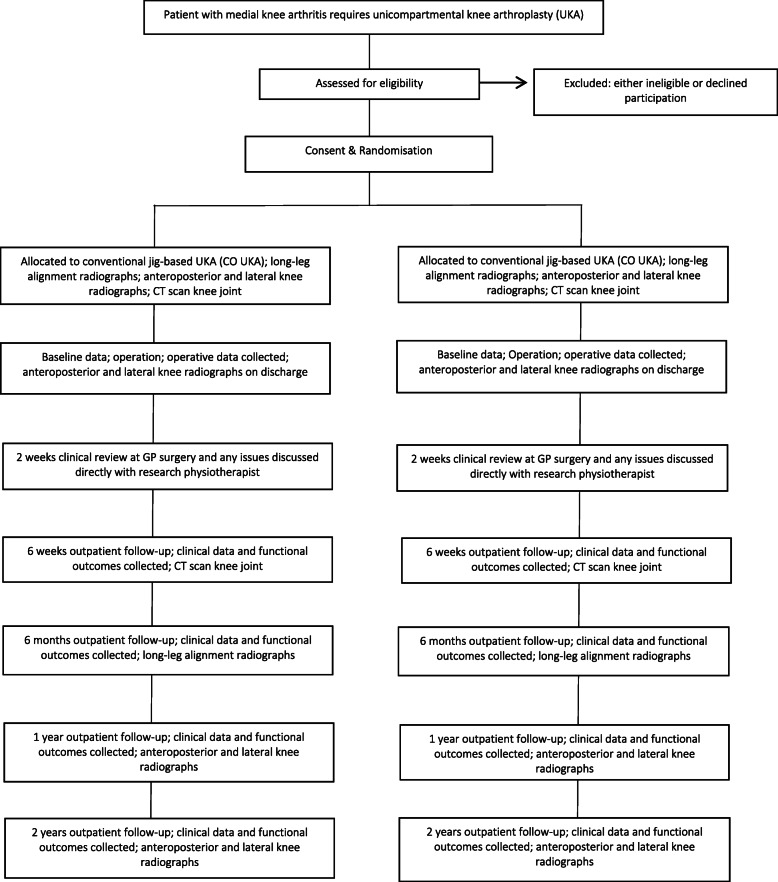
Patient enrolment flow chart

**Fig. 2:**
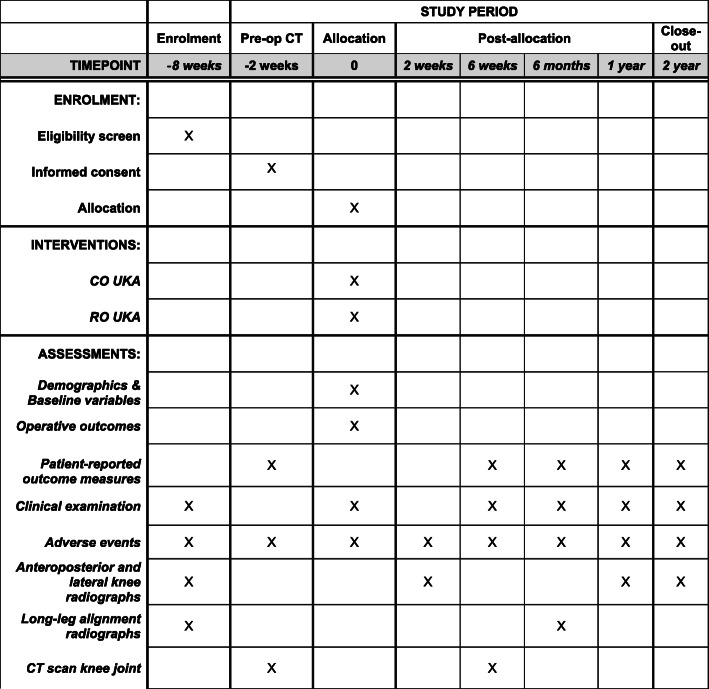
Schedule of enrolment, interventions, and assessments for all study patients. CO UKA, conventional jig-based unicompartmental knee arthroplasty; RO UKA, robotic-arm-assisted unicompartmental knee arthroplasty; CT, computerised tomography

This study is a prospective, single-centre, randomised controlled trial. The study will be undertaken in the Department of Trauma and Orthopaedics, University College Hospital, 235 Euston Road, Bloomsbury, London NW1 2BU, UK. The study will include 140 patients randomly allocated to either CO UKA (control group) or RO UKA (investigation group). The Oxford UKA was selected as the comparator as this was the most commonly used implant for UKA by all three operating surgeons at the study hospital and the most commonly used implant for UKA within the UK [1]. The study commenced patient recruitment in November 2017 and is expected to complete patient recruitment in April 2021. All patients will be followed up for 2 years after surgery, and therefore, the anticipated completion date for the study is April 2023. The study is sponsored by University College London, UK. The patient enrolment flowchart is presented in Fig. 1. The schedule of enrolment, interventions, and assessments for all study patients is shown in Fig. 2.

### Eligibility criteria

The inclusion criteria for this study are as follows: patient has symptomatic medial knee compartment arthritis requiring primary medial UKA, patient fit for surgical intervention following review by surgeon and anaesthetist, patient aged between 18 and 80 years at time of surgery, patient able to give informed consent and agrees to comply with the postoperative review programme, and patient has sufficient mobility to attend follow-up clinics. The exclusion criteria for this study are as follows: patient undergoing revision surgery following previously failed correctional osteotomy or ipsilateral UKA; patient not suitable for UKA (e.g. multi-compartment knee disease or ruptured anterior cruciate ligament); patient is immobile or has another neurological condition affecting musculoskeletal function; patient already enrolled on another concurrent clinical trial; patient unable or unwilling to sign the informed consent form specific to this study; and patient unable to attend the study follow-up programme.

### Recruitment

Patients will be recruited from the orthopaedic outpatient clinic at University College Hospital, London, UK. All patients will be screened by the clinical team (orthopaedic consultant surgeon, clinical research fellow, and orthopaedic registrar) for study participation based on the predefined inclusion and exclusion criteria listed above. Patients that fulfil the eligibility criteria and express an interest to participate in the study will be provided with an ethics committee-approved patient information sheet. This provides details about the study treatment, follow-up and contact details for further information. All members of the clinical team are familiar with the study and will address any preliminary questions about the study. Details of those patients expressing an interest to participate in the study will be recorded in the patient contact form and forwarded to the research physiotherapist. The research physiotherapist will phone the patient 4 weeks after this consultation to discuss any further questions and confirm if the patient would like to participate in the study.

### Consent

Informed consent will be obtained by the chief investigator or principal investigator when the patient attends for the preoperative planning CT scan. This is 6 weeks after the outpatient consultation for agreement to UKA and 2 weeks before surgery. It is important to the data collection scheme that patients are able to follow commands and read and interpret questions via questionnaires. For those who cannot hear, read, or understand English, an interpreter will be provided. Identical preoperative imaging modalities for surgical planning will be used in both treatment groups.

### Allocation

After informed consent has been obtained, the research physiotherapist will randomise the patient into one of the two treatment groups using an online random number generator (www.random.org). A number from 1 to 140 can be randomly generated and will allocate each patient to one of the two arms of the study: 1–70 inclusive for the control group, 71–140 inclusive for the investigation group. The research physiotherapist will perform the randomisation procedure and store the designated treatment group for each patient on a password-encrypted file on the hospital computer. The operating surgeon will have this information communicated to him on the morning of surgery.

### Preoperative imaging

All study patients will undergo preoperative long-leg alignment radiographs, anteroposterior and lateral knee radiographs, and CT scans of the knee joint. In both treatment groups, plain radiographs will be exported onto Traumacad software (Traumacad, Petach-Tikva, Israel) to template implant positioning and sizes for achieving the planned bone coverage, component position, and limb alignment. In CO UKA, fixed target values for component implantation will be obtained from the manufacturer’s manual, and the preoperative CT scans will be used to fine-tune bone resection and implant positioning. In RO UKA, preoperative CT scans will be exported onto a computer software programme (Mako system software, Stryker Limited, Kalamazoo, MI) to create a patient-specific, three-dimensional, computer-aided design model of the patient’s knee anatomy. This will be used to create a preoperative surgical plan for implant positioning. Intraoperative assessments of soft tissue tension, gap measurements, and limb alignment will be used to fine-tune bone resection and guide definitive component implantation. All preoperative templating will be undertaken by the senior supervising surgeon 2 weeks before surgery. Preoperative CT scans are not routinely used for preoperative surgical planning in CO UKA. However, both treatment groups in this study will have preoperative CT scans for three-dimensional surgical planning. This will help to limit any confounding effects from differences in preoperative planning techniques between the two treatment groups impacting the study outcomes.

### Surgical intervention

All surgical procedures will be performed under the direct supervision of a single surgeon using the minimally invasive medial parapatellar approach for medial UKA. A tourniquet will be applied, but not inflated unless there are intraoperative concerns with haemostasis. All patients will receive 1 g of tranexamic acid at induction. Patients in both treatment groups will receive 40 ml of 0.25% bupivacaine into the joint capsule prior to wound closure.

CO UKA will be performed using standard instrumentation, with extramedullary referencing to guide tibial bone resection and intramedullary referencing for femoral bone resection. Fixed target values will be used for all patients using the manufacturer’s recommendations and manual instrumentation. Tibial bone resection will be performed using a tibial saw guide positioned with its shaft parallel to the long axis of the tibia. A reciprocating saw with a narrow blade will be used to perform the vertical tibial cut medial to the origin of the ACL. An oscillating saw blade will be used to perform the horizontal tibial cut perpendicular to the mechanical axis of the tibia whilst matching the patient’s native posterior tibial slope. Extra-incisional bicortical femoral and tibial registration pins will be inserted prior to the medial parapatellar approach. Computer navigation will be used to assess knee kinematics and limb alignment before and after component implantation. Trials implants will be inserted and assessments of limb alignment, flexion-extension gaps, mediolateral laxity, and range of motion performed prior to definitive implant selection. The Oxford Phase-3 mobile-bearing cemented UKA (Zimmer Biomet, Bridgend, UK) will be implanted in all patients undergoing CO UKA.

RO UKA will be undertaken using extra-incisional bicortical femoral and tibial registration pins with fixed infra-red arrays mounted onto these to enable intraoperative optical motion capture technology to assess knee kinematics and alignment. Bone registration will be performed by mapping radiological landmarks displayed on the computer screen to register and verify osseous anatomy and bone geometry. Joint balancing will be used to capture femoral and tibial poses with corrective valgus and varus forces to assess knee kinematics through the arc of motion, and fine-tune implant positioning based on laxity of the soft tissue envelope. Bone resection will be performed within the stereotactic boundaries of the haptic bone windows using a high-speed, water-cooled burr with tactile, visual, and audio feedback. Optical motion capture technology will be used to assess limb alignment, flexion-extension gaps, mediolateral laxity, and range of motion with trial implants prior to definitive selection and cement implantation of final components. The RESTORIS MCK (Mako Surgical Corporation, Kalamazoo, Michigan) fixed-bearing UKA system will be implanted using the RIO robotic interactive arm orthopaedic system (Mako Surgical Corporation, Stryker, Kalmazoo, USA) in all patients undergoing RO UKA.

### Outcomes

All study patients will undergo review by two blinded observers (one orthopaedic registrar and one clinical research fellow) at 6 weeks, 6 months, 1 year, and 2 years following surgery. During these follow-up times, predefined clinical outcomes will be recorded by these observers using case report forms (CRFs). In addition, three independent observers (clinical research fellows) will collect radiological outcomes. It is not possible to blind observers recording radiological outcomes as different implant designs will be used within each treatment group. However, the three observers will independently calculate the accuracy of femoral and tibial implant positioning in each patient, and interobserver agreement for all radiological outcomes will be investigated using interclass correlation coefficients.

The following outcomes will be recorded in all study patients:

1. Accuracy of achieving the planned implant positioning as assessed using CT scans performed postoperatively at 6 weeks.
2. Operating time (minutes)
3. Time to hospital discharge (hours)
4. Analgesia requirements during inpatient admission and postoperatively at 6 weeks, 6 months, 1 year, and 2 years
5. Patient-reported outcome measures including Oxford knee score (OKS) and short form (SF-12), Western Ontario and McMaster Universities Arthritis Index (WOMAC), Knee injury and Osteoarthritis Outcome Score (KOOS), and University College Hospital (UCH) functional score during inpatient admission and postoperatively at 6 weeks, 6 months, 1 year, and 2 years.
6. Health-related quality of life as measured using European Quality of Life questionnaire with 5 dimensions for adults (EQ-5D) preoperatively and postoperatively at 6 weeks, 6 months, 1 year, and 2 years
7. Mobilisation distance (metres) and use of mobility aids during inpatient admission and postoperatively at 6 weeks, 6 months, 1 year, and 2 years
8. Range of movement (degrees) in the knee joint during inpatient admission and postoperatively at 6 weeks, 6 months, 1 year, and 2 years
9. Resource use and cost-effectiveness including comparisons between the two treatment groups relating to: Operating time, theatre efficiency, equipment and sterilisation costs, analgesia requirements, inpatient rehabilitation, time to discharge, outpatient follow-up, additional imaging costs, and need for further surgery.
10. Complications

All patients will undergo preoperative and postoperative CT scans of the knee joint using a standardised protocol within a dedicated research scanner in the study hospital. The CT scans will be uploaded in DICOM (Digital Imaging and Communication in Medicine) format and then loaded onto Mimics software (Materialise) to calculate the accuracy of implant positioning. Accuracy of implant positioning will be assessed by comparing differences in the target values in the preoperative plan to the achieved values in the postoperative scan. These outcomes will be used to calculate root mean square errors values for accuracy of femoral and tibial component positioning within the coronal, sagittal, and axial planes as described by Bell et al. [4]. The posterior condylar offset ratio (PCOR) and posterior tibial slope will be assessed using the methods described by Gaudiani et al. and Johal et al. respectively [13, 23].

The WOMAC, OKS, SF-12, EQ-5D, and KOOS are validated tools for the clinical assessment of patients after knee arthroplasty [11, 12, 17, 22, 39]. In addition, the observers will calculate the UCH knee score to assess overall, pain, function, and mobility [19]. This will help to overcome any potential ceiling effect with the OKS and WOMAC and facilitate further subgroup analysis in patients with high functional demands [19]. Each of these clinician- and patient-reported scores will be collected preoperatively at the time of consenting to the study and also postoperatively at 6 weeks, 6 months, 1 year, and 2 years after surgery.

### Blinding

All patients and clinical staff clinical outcomes will remain blinded to the treatment group. It is not possible to blind observers recording radiological outcomes as different implant designs will be used within each treatment group. Both conventional manual instrumentation and robotic-arm-assisted surgery are only compatible with implants from their respective manufacturers, and so identical implant designs could not be used in both treatment groups. Study patients will be identifiable with a unique study number. Only the research physiotherapist will have the key to identify individual patients and their respective treatment arm. Any documents related to the study will be archived directly at the study site by the research physiotherapist within a locked filing cabinet in a locked research office. This office has swipe card access with onsite security and 24-h CCTV surveillance. Patient data will be logged electronically using each patient’s unique identification number with computer software on an encrypted, password-protected research computer.

### Sample size

Using the study by Bell et al. assessing differences in accuracy of component positioning between conventional and robotic UKA, the mean difference in femoral sagittal component positioning was set at 2° and standard deviation assumed at 4° [4]. Using a two-tailed, two-sample *t* test with a power of 80% (1–β), significance level of 5%, and an effect size of 0.5, this study required 128 patients to detect this minimum difference between the two treatment groups. To account for 10% attrition in the sample size during follow-up, the sample size was set at 140 patients.

### Statistical analysis

The analysis of the per-protocol population will be considered the primary analysis. The differences between the CO UKA and RO UKA groups will be analysed by calculating the difference from baseline, per patient, and a two-sided confidence interval for the difference between the changes from baseline values will be calculated. This confidence interval will cover the true difference in the percentage change from baseline with a probability of 95%. The following statistical methods will be employed to analyse the data: descriptive statistics, independent *t* test, paired *t* test, analysis of variance, Fisher’s exact test, chi-square test, and graphical displays. Assumptions of normality will be tested with the D’Agostino test. Assumptions of homogeneity of variance will be tested with Levene’s test. If the distributional assumptions are (severely) violated, non-parametric techniques, such as Mann-Whitney’s test, will be employed. In the event that RO UKA is converted to CO UKA intraoperatively, analysis will be performed using the intention-to-treat population and the treatment actually received by the patients. Intraoperative conversion from RO UKA to CO UKA will be documented and presented and published as part of the study. The Bonferroni correction will be used to determine the level of significance due to multiple comparisons of secondary outcomes. Statistical significance is set at a *p* value < 0.05 for all analyses, and all statistical analysis will be performed using SPSS software version 26 (SPSS Inc., Chicago, IL, USA).

### Adverse events

Adverse events are defined as any untoward medical occurrence in a patient or study participant, which does not necessarily have a causal relationship with the procedure involved. A serious adverse event (SAE) is an adverse event that results in hospitalisation or prolongation of existing hospitalisation, persistent or significant disability or incapacity, life-threatening clinical sequelae, or death. All SAEs during the protocol treatment will be reported directly to the sponsor using the SAE web form. The chief investigator will also assess the SAE for severity, causality, seriousness, and expectedness using pre-existing criteria provided by the sponsor and inform the Data Safety Monitoring Board (DSMB) within 3 days of the initial observation of the event. The protocol treatment period is defined as the period from the day that the first study patient is recruited into the trial to the day that the final study patient has completed 2 years follow-up. The chief investigator will also inform the London-Bloomsbury Research Ethics Committee and local Health Research Authority within 3 days of the SAE taking place. Safety aspects of the study are closely monitored by the sponsor and DSMB using unblinded data for its judgement. In cases where the SAE arises due to a problem with the robotic device, Stryker Limited will also be notified within 2 days of the event taking place. The chief investigator will record the following: onset date, complete description of the event, severity, duration, action taken, and outcome for each SAE. The chief investigator will also provide regular updates of all SAEs to the London-Bloomsbury Research Ethics Committee, Local Health Research Authority, DSMB, and sponsor.

### Data management

On-site monitoring visits shall occur throughout the course of the clinical study by the chief investigator. The chief investigator shall permit and assist the sponsor (should they choose to monitor the study) to carry out verification of all study forms against data in the source documents, which shall occur as per the departmental policy for undertaking such activities. University College Hospital recognises that there is an obligation to archive study-related documents at the end of the study. The study master file will be archived at University College London in accordance with the University College Hospital Standard Operating Procedure for Archiving of Investigator Site File (ISF) and Pharmacy Site File (PSF). It will be archived for a minimum of 5 years from the study end and no longer than 30 years from the study end.

### End of protocol treatment

Reasons for going off study protocol include:

- Completion of last follow-up visit 2 years after surgery
- Patient non-compliance or withdrawal (the reason for discontinuation will be recorded in the case report form)
- Intercurrent death

All patients included into this study are free to withdraw from the study at any time without compromise to their future treatment. On withdrawal, patients will revert to the standard follow-up regimen for routine (non-study) UKA at the study site. The end of study form will be completed and the reason for withdrawal documented. This form will also be completed if the patient is lost to follow-up or dies during the course of the study. Data to the point of discontinuation will be used for analysis.

### Monitoring

The chief investigator will monitor the progress of the clinical study in the form of monthly research meetings for those involved in the trial. The chief investigator will be responsible for day to day monitoring and management of the study. The UCLH/UCL/Joint Research Office, on behalf of UCL as Sponsor, will monitor and conduct random audits on a selection of studies in its clinical research portfolio. Monitoring and auditing will be conducted in accordance with the Department of Health Research Governance Framework for Health & Social Care (April, 2005) and in accordance with the sponsor’s monitoring and audit policies and procedures. As per the protocol, the principal investigator will email the sponsor twice yearly with the following: delegation log, adverse event log, deviation log, and any annual progress reports sent to the Ethics committee.

### Peer review

The study protocol has undergone independent external peer review. The suggestions and recommendations for improvement to the study design were implemented. The reviewers, sponsor, and London-Bloomsbury Research Ethics Committee reviewed the revised protocol documents and confirmed that all queries and suggestions had been fully addressed.

## Discussion

Accuracy of component positioning and limb alignment are important prognostic variables that affect implant survival and time to revision surgery following UKA [5, 9, 38]. CO UKA is performed using manually positioned alignment guides, limited intraoperative data on knee kinematics, and handheld milling devices or sawblades for bone resection. However, these manual techniques are highly dependent on the skill and expertise of the operating surgeon. Surgeon-induced errors in implant positioning are the leading cause of premature implant failure and early revision surgery following UKA [29–32]. RO UKA uses a preoperative CT scan to create a virtual three-dimensional reconstruction of the patient’s osseous anatomy. The surgeon uses this virtual model to plan optimal bone coverage, implant positioning, and limb alignment. An intraoperative robotic arm then helps to execute this plan with a high-level of accuracy and reproducibility, and stereotactic boundaries limit bone resection to the predefined femoral and tibial haptic windows [4, 10, 26]. This prospective randomised controlled trial will include 140 patients with symptomatic medial compartment knee arthritis undergoing primary UKA. Following informed consent, patients will be randomised to CO UKA (control group) or RO UKA (investigation group) at a ratio of 1:1 using an online random number generator. Observers will review patients at regular intervals for 2 years after surgery to record predefined study outcomes pertaining to accuracy of implant positioning, limb alignment, postoperative rehabilitation, clinical progress, functional outcomes, cost-effectiveness, and complications. The following statistical methods will be employed to analyse the data: descriptive statistics, independent *t* test, paired *t* test, analysis of variance, Fisher’s exact test, chi-square test, and graphical displays. The findings of this study will provide an improved understanding of the differences in CO UKA versus RO UKA with respect to accuracy of implant positioning, limb alignment, patient satisfaction, functional outcomes, implant survivorship, cost-effectiveness, and complications.

## Trial status

Protocol: version 1.0; date 18 April 2017.

Patient recruitment date: 1 November 2017.

Estimated completion of recruitment date: 1 April 2021.

Estimated completion of final follow-up: 1 April 2023.

## Data Availability

The datasets used and/or analysed during the current study are available from the corresponding author on reasonable request.

## Abbreviations

CO UKA: Conventional jig-based unicompartmental knee arthroplasty
CT: Computerised tomography
DSMB: Data Safety Monitoring Board
EQ-5D: European Quality of Life questionnaire with 5 dimensions for adults
KOOS: Knee injury and osteoarthritis outcome score
OKS: Oxford knee Score
RO UKA: Robotic-arm assisted unicompartmental knee arthroplasty
SAE: Serious adverse event
SF-12: Short-form health survey of 12 items
UCH: University College Hospital knee functional score
WOMAC: Western Ontario and McMaster Universities Arthritis Index
